# Faults Diagnostics of Railway Axle Bearings Based on IMF’s Confidence Index Algorithm for Ensemble EMD

**DOI:** 10.3390/s150510991

**Published:** 2015-05-11

**Authors:** Cai Yi, Jianhui Lin, Weihua Zhang, Jianming Ding

**Affiliations:** State Key Laboratory of Traction Power, Southwest Jiaotong University, Chengdu 610031, China; E-Mails: lin13008104673@126.com (J.L.); tpl@swjtu.edu.cn (W.Z.); fdingjianming@126.com (J.D.)

**Keywords:** ensemble empirical mode decomposition, Hilbert transform, axle bearing, fault diagnostics, intrinsic mode function, marginal spectrum

## Abstract

As train loads and travel speeds have increased over time, railway axle bearings have become critical elements which require more efficient non-destructive inspection and fault diagnostics methods. This paper presents a novel and adaptive procedure based on ensemble empirical mode decomposition (EEMD) and Hilbert marginal spectrum for multi-fault diagnostics of axle bearings. EEMD overcomes the limitations that often hypothesize about data and computational efforts that restrict the application of signal processing techniques. The outputs of this adaptive approach are the intrinsic mode functions that are treated with the Hilbert transform in order to obtain the Hilbert instantaneous frequency spectrum and marginal spectrum. Anyhow, not all the IMFs obtained by the decomposition should be considered into Hilbert marginal spectrum. The IMFs’ confidence index arithmetic proposed in this paper is fully autonomous, overcoming the major limit of selection by user with experience, and allows the development of on-line tools. The effectiveness of the improvement is proven by the successful diagnosis of an axle bearing with a single fault or multiple composite faults, e.g., outer ring fault, cage fault and pin roller fault.

## 1. Introduction

Fault diagnosis based on the analysis of vibration signals has shown a great development, based on the characterization of mechanical system condition and allowing early detection of a possible fault. Whatever the mechanical system, the evaluation of both type and the fault position allows the reduction of the plant standstill time. Therefore, from an industrial point of view, a proper diagnostic approach reduces both the time and the costs required for repairs [1,2,3]. Rail vehicle axle-bearings are one of the most important components of railway vehicles regarding transportation safety. Due to all the factors such as design, installation technology, use conditions and sudden load, it is hard for railway axle bearings to avoid wear, pitting, crack, stripping and abrasion and so on. These failures are serious issues, often resulting in service delays and potentially fires and derailments, with obvious risks to life [4,5]. In July 2008, an ICE3 high speed train rated for 330 km/h service speed derailed during departure from Cologne, Central Station, Germany, due to the fatigue failure of one of the driving axles. It is shown that a “dead” train on the railway line has implications for customer perception, operator and manufacturer reputation, along with large costs from penalties, recovery, train availability and repairs [6,7,8].

In recent years, railways have experienced significant changes in the vehicle-track interaction, caused by the increase in axle load and operational speed. The advent of high speed traffic on railways has increased the importance of avoiding hot axle bearings. The dangers of overheating of axle bearings of railway coaches in motion has necessitated the provision of means of early warning. Various technologies and approaches have been adopted to continuously monitor the state of axle bearings. Hot axle box detection (HABD) has been used in the UK national rail network for decades as a safety requirement. The HABD is sited either on-train for inboard or high-speed bearings, or trackside for outboard axle-boxes. Approximately 220 trackside HABD detectors are currently installed in the UK [9]. A bearing that is creating sufficient heat to trigger an HABD will already be in full failure mode. Therefore, the train has to be stopped immediately causing disruptions to passengers and railway traffic. For train operators in the UK, two approaches to degradation diagnostics are currently available: one is the trackside system, e.g., Railway Bearing Acoustic Monitoring, Trackside Acoustic Detection System developed by the American Transportation Technology Centre, both employing microphones to listen to passing wheel-set bearings [9], or “black-body” radiation, which is used for temperature measurement by infra-red detectors [10]; the other is the on-board system developed by Perpetuum Ltd. [11]. The latter approach (the subject of this paper) provides local real-time vibration monitoring of each wheel bearing using accelerometers. In China, the Axle Temperature Alarming Device (ATAD), as an important vehicle monitoring instrument, that must be installed on each railway passenger vehicle, and ensures all being always working and available [12], which is also on-board detection. Neither method of detection (track-side or on-board) will prevent early bearing degradation, but each will alert the operators so early intervention (before HABD) is possible, preventing catastrophic outcomes and reducing maintenance costs.

Using the on-board condition monitoring sensor, an operator can not only look for changes in vibration/temperature, but also evaluate both the fault type and the fault position, which is of direct engineering significance. There are various signal processing approaches in fault diagnostics, which are based on different theoretical backgrounds, and also the results obtained are often different. Some techniques may be more suitable than others for a specific system or component, depending also on the environmental conditions. Therefore, it is important to choose an approach that is the most effective for the case and the situation under testing for a reliable mechanical analysis.

An advanced fault diagnostics method suitable for railway axle bearings in general should accomodate non-stationary and non-periodic vibration signals, meaning that the analysis is based intrinsically on data, and suitable for on-line diagnosis. The empirical mode decomposition (EMD) and the following Hilbert transform (HT) and marginal spectrum belong to this kind of methods. The EMD presented in 1998 by Huang *et al.* [13] is a signal processing method appropriate for analyzing nonlinear and non-stationary signals. Differently from other transformations, EMD is an adaptive decomposition, and no hypothesis about signal periodicity or stationary must be respected for EMD applications. The EMD method is developed from the simple assumption that any signal consists of different simple intrinsic modes of oscillations, which is useful for the extraction of the intrinsic mode functions (IMFs), or monocomponent functions, composing the original signal, while HT of the extracted functions is used for the instantaneous frequency evaluation. The major drawback of the original EMD is the mode mixing [14,15], which is the consequence of signal intermittence. The intermittence could cause the aliasing problem and makes the physical meaning of the IMF unclear. To overcome the mode mixing separation problem, a new noise-assisted data analysis (NADA) method was proposed by Wu and Huang [16]. This method is named the Ensemble EMD (EEMD) [16]. It defines the true IMF components as the mean of an ensemble of trials, each consisting of the signal plus white noise of finite amplitude. Compared with EMD, EEMD is more accurate and effective for rotating machinery fault diagnosis [17,18,19,20].

Mahgoun *et al.*, presented a brief description of the algorithm used to get the residual signal from the EEMD method, and the residual signal is obtained by removing some IMFs which represent the noise; Kurtosis is used in the measurement of impulsiveness, and signals that have a Kurtosis less or equal to 3 were eliminated [2]. Ricci *et al.*, used the merit index, EMD and HT to diagnose gear faults and a merit index was introduced that allows the automatic selection of the intrinsic mode functions. The merit index as a matter of fact is a linear combination of two indexes: the first is a measure of the periodicity degree of the IMF, while the second one is represented by its absolute skewness value [3]. Yan *et al.*, presented a weak signal detection methodology based on the improved Hilbert-Huang transform, and replaced the original signal with a residual signal by reconstructing IMFs from the denoised detail coefficients and approximated coefficients through the inverse wavelet transform [21]. Eftekhar *et al.*, utilized sliding overlapped windows to execute the EMD algorithm in real-time [22]. A review of the available literature on EMD algorithms demonstrates a need for a local and online method. This paper thus aims to propose such an improved ensemble EMD algorithm for real-time applications. It is an idea-screening process for building IMFs’ confidence index to discriminate axle bearing faults, which is adaptive and automatic. HT is then applied to IMFs selected by the confidence index to obtain the corresponding Hilbert spectrum, that is, these IMFs are expressed in the time-frequency domain, and then these IMFs’ Hilbert spectrum will be aggregated to order marginal spectrum derived in the feature extraction stage. Using the IMFs’ confidence index and HT marginal spectrum become an advanced fault diagnostics tool having the characteristic previously listed with a high degree of automation and self-adaption.

The rest of this paper is organized as follows: in Section 2, the ensemble EMD algorithm, HT and its marginal spectrum are reviewed. Section 3 presents the IMFs’ confidence index algorithms; In Section 4 shows the experimental test. The algorithms application and experimental results are shown and discussed in Section 5. The conclusions about the effectiveness and the diagnostics capability of the method are reported in Section 6.

## 2. Basic Theory of EEMD and Marginal Spectrum

### 2.1. Principles of EMD and EEMD

A quick review of the EMD algorithm is conducted based on [13]. The EMD algorithm includes two loops. The inner loop is called the sifting process. The main task of the sifting process is to view the investigated signal as the approximation and detail parts, and to separate them from each other. Thanks to the definition of the interpolating splines, the extraction of a mean function *m*(*t*) is possible and it can be removed from the initial signal *x*(*t*) in order to obtain:

(1) $$x_{1}(t)=x(t)-m(t)$$

The obtained signal *x*_1_(*t*) is now examined with the aim to evaluate if it respects the intrinsic mode functions (IMF) definition. In this way, it can smooth uneven signals, and each signal could be decomposed into a number of IMFs, each of which must satisfy the following definition [13]: in the whole data set, the number of extreme and the number of zero-crossings must either equal or differ at most by one; at any point, the mean value of the envelope defined by local maxima and the envelope defined by the local minima is zero.

An IMF represents a simple oscillatory mode compared with the simple harmonic function. If the two previous conditions are not satisfied, the resulting signal *x*_1_(*t*) is not an IMF, and the previous checks are repeated. The sifting process runs until the extracted signal respects the two IMF conditions; then the function obtained represents the first intrinsic mode function *c*_1_(*t*) and it is subtracted from the initial signal:

(2) $$r_{1}(t)=x(t)-c_{1}(t)$$

where *r*_1_(*t*) is the residual signal, which represents the input for the second IMF calculation by means of the sifting process. The EMD algorithm, applied to the original *x*(*t*), stops when the residual signal *r_n_*(*t*) is a constant or monotonic function. From the above and with the definition, any signal *x*(*t*) can be decomposed as:

(3) $$x(t)=\sum_{i=1}^{n}c_{i}+r_{n}(t)$$

The original signal can be expressed as the sum of all the IMFs and the residue. The IMFs include different frequency bands ranging from high to low. EEMD’s procedures are as follows [16,17,18,19,20]:

(1) Add a random white noise signal *n_j_*(*t*) to *x*(*t*).
  (4) $$x_{j}(t)=x(t)+n_{j}(t)$$
  where *x_j_*(*t*) is the noise-added signal, *j* = 1, 2, 3, …, *M*, and *M* is the number of trials.
(2) Decompose *x_j_*(*t*) into a series of intrinsic mode functions *c_i,j_* utilizing EMD as follows:
  (5) $$x_{j}(t)=\sum_{i=1}^{N_{j}}c_{ij}+r_{N_{J}}$$
  where *c_ij_* denotes the *i*th IMF of the *j*th trial, *r_Nj_* denotes the residue of *j*th trial and *N_j_* is the IMFs number of the *j*th trial.
(3) If *j* < *M*, then repeat steps 1 and 2, and add different random white noise signals each time.
(4) Obtain *I* = min(*N*_1_, *N*_2_, …, *N_M_*) and calculate the ensemble means of corresponding IMFs of the decompositions as the final result
  (6) $$c_{i}=\left(\sum_{j=1}^{M}c_{ij}\right)/M$$
  where *i* = 1, 2, 3, …, *I*; *c_i_* (*i* = 1, 2, 3, …, *I*) is the ensemble mean of corresponding IMF of the decompositions.

### 2.2. Hilbert Transform and Hilbert Marginal Spectrum

For each IMF *c_i_*(*t*), we can obtain its Hilbert transform, and *f*(*t*) can be expressed by convolution of *f*(*t*) and 1/π*t*. In order for the numerical implementation, the discrete form $\frac{1-（-1{）}^{t}}{\pi t}$ is used instead of 1/π*t*, where *t* = 1, 2, …, *M*. Then the Hilbert transform of *c_i_*(*t*) can be expressed as:

(7) $${\hat{c}}_{i}(t)=c_{i}(t)*\frac{1-{\left(-1\right)}^{t}}{\pi t}=\frac{2}{\pi }\sum_{k=0}^{M-1}\frac{c_{i}\left(t-2k-1\right)}{2k-1}$$

Thus the analytical signal of the original signal is obtained by:

(8) $$z_{i}(t)=c_{i}(t)+i{\hat{c}}_{i}(t)=a_{i}(t)e^{j\theta_{i}(t)}$$

Instantaneous frequency is expressed by:

(9) $$\omega_{i}(t)=\frac{d\theta_{i}(t)}{d(t)}$$

After performing the Hilbert transform on each IMF component, the original signal can be expressed as the real part (Re) in the following form:

(10) x(t)=∑i=1nci(t)=Re∑i=1nzi(t)=Re∑i=1nai(t)ejθi(t)=Re∑i=1nai(t)ej∫0Tθi(t)dt

Here we left out the residue *r_n_* on purpose, for it is either a monotonic function or a constant. Meanwhile, for the same signal *x*(*t*), the Fourier expansion can be expressed as:

(11) $$x(t)=\sum_{i=1}^{\infty }a_{i}e^{j\theta_{i}t}$$

From Equations (10) and (11), it is shown that the Fourier transform is a special form of the HT. Amplitude variation and instantaneous frequency not only improve the effectiveness of decomposition significantly, but also make HT based on EEMD suitable for non-stationary signals. The transformations of amplitude and frequency can be clearly separated by using each IMF component’s expansion, which mitigates Fourier transform’s limitation in terms of invariable amplitude and frequency [23]. The time-frequency-amplitude distribution is designated as the signal’s Hilbert spectrum *H*(ω, *t*), which can accurately describe amplitude changes with time and frequency and further reflect the signal’s inherent time-varying characteristics. With the Hilbert spectrum defined, the Hilbert marginal spectrum can be shown as:

(12) h(ω)=∫−∞+∞H(ω,t)dt=∫−∞+∞Re∑i=0nai(t)ej∫0Twi(t)dtdt

Obviously, the Hilbert spectrum offers a measure of amplitude distribution from each frequency and time, while the marginal spectrum gives a measure of the total amplitude distribution from each frequency.

## 3. IMFs’ Confidence Index Algorithms

EEMD algorithms adaptively decompose the signal into different simple intrinsic modes of oscillations which are extracted to form the IMFs, and then the choice of the IMFs actually is a process to filter and reduce noise in the signal. In general, the choice of the IMFs to be analyzed is realized by means of visual or experience criteria of the user. In this way, for all that the ensemble EMD arithmetic is fully adaptive, the subsequent calculation process cannot continue to be automatic and some interaction with the user is required. This becomes the key limitation of practical applications. If the arithmetic can build up the confidence of IMFs independently, and those IMFs which are worthwhile and meritorious for fault diagnosis are automatically selected, it would be a major breakthrough to solve practical problems in engineering.

**Figure 1:**
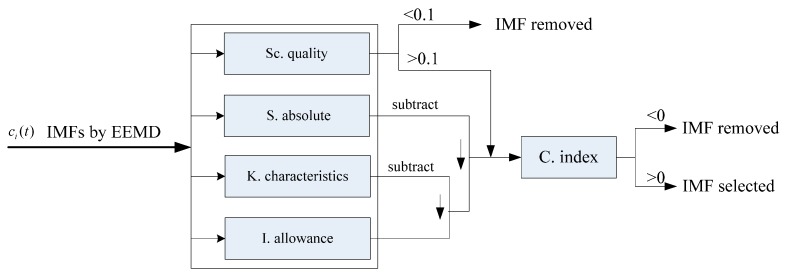
IMFs’ confidence index algorithm computational procedure.

To make the EEMD and calculations faster and automatic, a confidence (C.) index is introduced. The implemented index, as a matter of fact, is a computational procedure, shown as Figure 1.

There are four indexes involved in the arithmetic. The first is a measure of the self-correlation degree of each IMF, named self-correlation (Sc.) quality. The Sc. quality is represented by the standard deviation of the self-correlation coefficient to state the interaction of the data set, which is calculated by Equation (13). The second index is represented by the absolute skewness value of each IMF, named skewness (S.) absolute. Skewness is to detect the asymmetry of the probability distribution of the data set, which may be positive or negative, but the size is the focus to measure, so S. is calculated by Equation (14). Kurtosis is one dimensionless parameter to reflect the distribution features of vibration signals, and is particularly sensitive to impact signals. The square of the reciprocal of kurtosis is used to measure the fault feature visibility of each IMF in the IMF’s confidence index algorithm, and this index is called kurtosis (K.) characteristics, represented by Equation (15). Finally the fourth index also concerns the impact in signala, used to measure the degree of influence of impact responses and named Impact (I.) allowance. The impact points in this paper are the maximum value points exceeding the square root of the maximum value envelope mean for each IMF, and the reciprocal of the standard deviation of two consecutive impact points with respect to wheel rotation period is defined as the I. allowance shown in Equation (16):

(13) $$Sc.quality=\sigma (r_{c_{i}(t),c_{i}(t-1)})$$

where $r_{c_{i}(t),c_{i}(t-1)}$ is the self-correlation coefficient of each IMF:

(14) $$S.absolut=\left|Skew\left(c_{i}\right)\right|$$

(15) $$K.characteristics={\left[1/kurtosis(c_{i})\right]}^{2}$$

If the number of detected maximum values *i.e.*, impact points (IP) is *l*, and the wheel rotational period is *T*, then:

(16) $$I.allowance=\frac{1}{\sigma \left(\frac{IP_{l+1}-IP_{l}}{T}\right)}$$

If there is much noise in a signal, the relevance of the data set is inevitably small, then the IMF containing the fault signature generally has a high correlation degree. The standard deviation of an IMF’s self-correlation coefficient represents the possibility of a fault feature existing, and the evaluation threshold value for the experimental cases presented here is 0.1: if the IMF is less-than the threshold value, it would be removed; otherwise it is selected for further calculations automatically. When a fault appears in a bearing, the impact pulse will be generated by the fault in the rotation period. The greater the fault, the larger the amplitude of the shock response is and the more obvious the failure phenomenon is. The kurtosis coefficient represents the probability of failure formation of the appeared large amplitude pulse, while the square of the reciprocal of the kurtosis coefficient is a measure of the fault feature visibility, which is implemented to subtract the Impact allowance to obtain the visibility of the fault feature’s essential characteristics. To appraise the Impact allowance, the IMF envelope is calculated to obtain its impact points exceeding the extraction of the root of the envelope average. The differences between the abscissas of two consecutive impact points are then evaluated in terms of number of points. Then the differences normalized with respect to wheel rotation period make up a set of data. The reciprocal of the standard deviation of the resulting data set represents the impact response influence degree of the IMF. Since no information about data set symmetry is taken into account by I. allowance and K. characteristics, skewness of the IMF is calculated in order to check the distribution of the data set about the zero value. Since high absolute skeness values can be obtained for asymmetric data sets or high impact influence time series, then the IMFs’ confidence index expressed in Figure 1 assumes positive values for containing fault signature IMFs.

The IMF selection is very related with the confidence index: one IMF is transformed with HT if it is not removed by the index arithmetic computational procedure. It is worth noting that the whole arithmetic computational procedure does not require any assumptions and empirical estimation, and is quite fully automated and adaptive.

## 4. Experimental Tests

**Figure 2:**
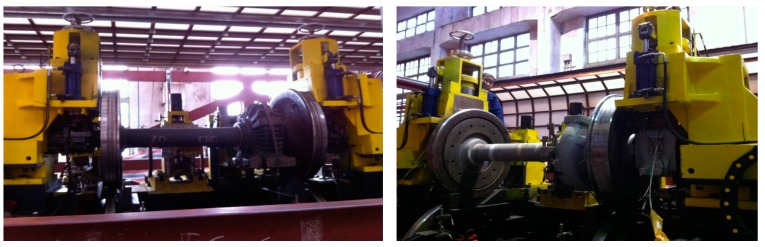
Railway axle bearing test-rig.

**Figure 3:**
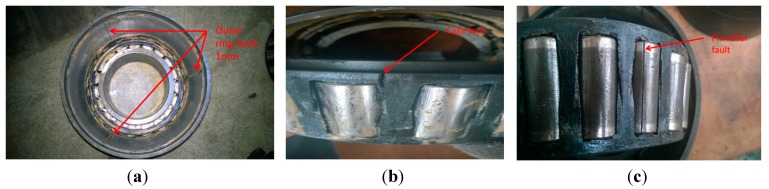
Axle bearing parts with different faults: (**a**) outer ring fault; (**b**) cage fault; (**c**) pin roller fault.

An experiment test on railway axle bearings was performed in the test-rig shown in Figure 2. It can control the motor rotation speed, *i.e.*, the wheel speed, and the axle-box vibration acceleration is collected through an IMC data acquisition system using a strain sensor with a sample frequency of 10 kHz. The failure parts of railway axle bearings contain outer ring faults, cage faults and pin roller faults. These failure parts were emphasized and re-introduced artificially based on the original and weak faults of these axle bearings generated during in-service train: there are three grooves ablated with depth of 1 mm on the inner surface of the bearing outer ring presenting a 120°-angle distribution as shown in Figure 3a, and there is a fracture on the bearing cage between rollers in Figure 3b. Figure 3c shows the pin roller ablated fault with a depth of 1 mm.

Multiple experimental schemes are designed, including single type of fault experiments, and two or three types of fault combination tests. Different working conditions have been considered: the experimental tests have been carried out at the different wheel speeds and the different categories of failures are included. The local damage occurs in the rolling bearing, which will produce the corresponding vibration frequency, referred to as the fault characteristic frequency. The characteristic frequency of each kind of fault is always different from others. Since the axle bearing is characterized by a smooth behavior due to the high contact ratio, defects could be hidden during the operation and their detection allows evaluating the effectiveness of EEMD and IMFs’ confidence index algorithms.

For fault diagnosis of railway axle bearing, the fault characteristic frequency of the bearing vibration signal is the most intuitive and direct analysis method. The bearing vibration signal is quite informative and complex sampled by sensors, so the fault characteristic frequencies of the bearing are calculated in advance:

(17) $$f_{i}=\frac{n}{60}$$

(18) $$f_{a}=\frac{1}{2}Z(1-\frac{d}{D}\cos\alpha )f_{i}$$

(19) $$f_{b}=\frac{1}{2}(1-\frac{d}{D}\cos\alpha )f_{i}$$

(20) $$f_{c}=\frac{D}{2d}\left[1-{\left(\frac{d}{D}\right)}^{2}\cos^{2}\alpha \right]f_{i}$$

According to Equations (17)–(20), the characteristic frequencies of a single bearing fault would be calculated. The *f_i_*, *f_a_*, *f_b_* and *f_c_* represent the rotation frequency of the bearing inner ring, outer ring fault characteristic frequency, cage fault characteristic frequency and pin roller fault characteristic frequency, respectively. In Equations (17)–(20), *n* is the inner ring speed of bearing, *d* is the diameter of roll, *D* is the average value of the inner ring and outer ring diameter, *Z* is the number of rolling element, and α is the pressure angle (contact angle). The actual calculated values of fault characteristic frequencies when the wheel experiment speed is 100 km/h are shown in Table 1. It is worth noting that just one fault’s corresponding characteristic frequency is acquired by Equations (18)–(20). Under the same fault condition, if there is more than one damage point, the corresponding fault characteristic frequency is a multiple of the single fault characteristic frequencies, and the multiple size is determined by the number of damage points, e.g., there are three damage points on the bearing outer ring, so the fault characteristic frequency of the outer ring fault for the axle bearing is 3 × *f_a_*.

**Table 1:** Axle bearing characteristic frequencies at a wheel experiment speed of 100 km/h.

Axle Bearing Parameter and Faults	Fault Characteristic Frequency (Hz)
Rotation frequency of inner ring (*f_i_*)	10.2865
Outer ring fault (*f_a_*)	83.2979
Cage fault (*f_b_*)	4.3898
Pin roller fault (*f_c_*)	33.9294

## 5. Algorithm Applications

**Figure 4:**
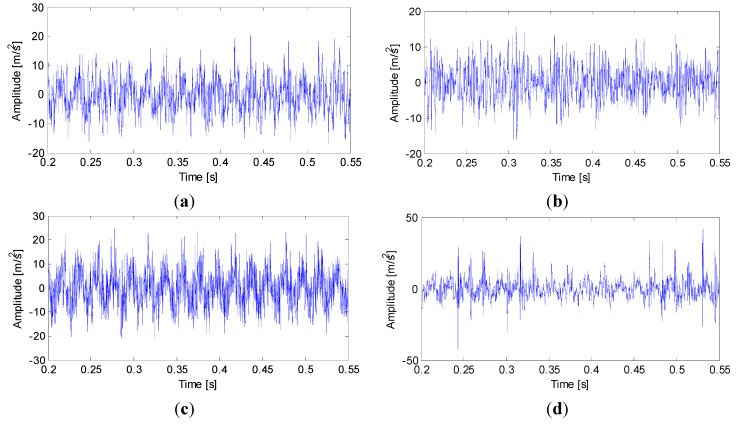
Axle-box vibration signals with undamaged and damaged axle bearings: (**a**) undamaged bearing; (**b**) outer ring fault bearing; (**c**) cage fault bearing; (**d**) pin roller fault bearing.

The algorithm applications for axle-box vibration signals acquired by means of the vertical accelerometer for the undamaged and damaged axle bearing are reported respectively in Figure 4a–d. The wheel runs at a speed of 100 km/h for a period of two minutes for each working condition. Only an arbitrary portion of the complete acquired signal, lasting 0.35 s, is analyzed. By comparing the four time histories, it is easy to note that the highest and sudden variations of vibration occur when the pin roller fault bearing happens, and the most cyclical and regular variation of vibration occurs in cage fault bearing vibrations. The former fact can be explained by the different alignment conditions during the tests, or the essential characteristics of the fault similar with the latter fact, which occurs at the damage point. The simple comparison of the four signals in time-domain is misleading and this makes it difficult to evaluate the axle bearing state.

**Figure 5:**
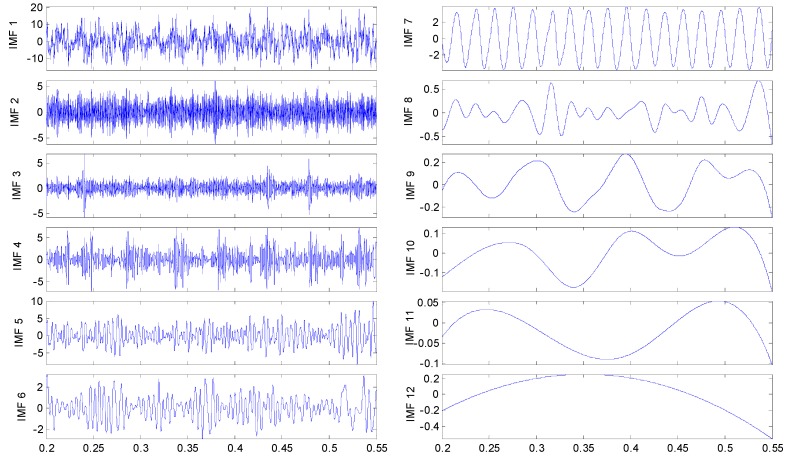
IMFs for the undamaged axle bearing vibration signal.

**Figure 6:**
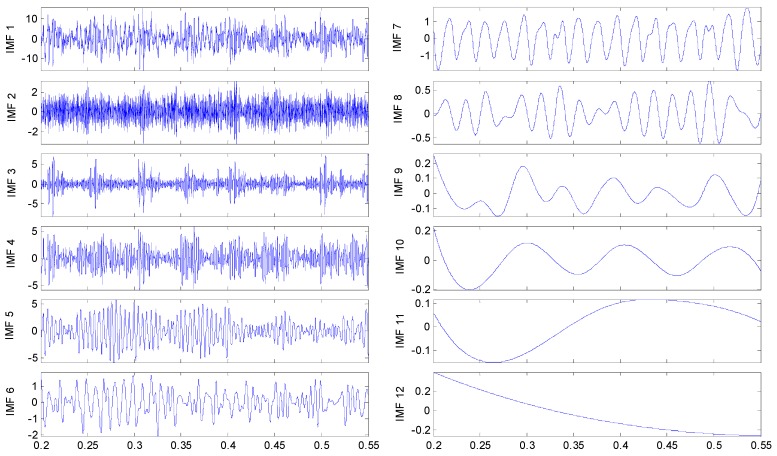
IMFs for the axle bearing vibration signal with outer ring fault.

**Figure 7:**
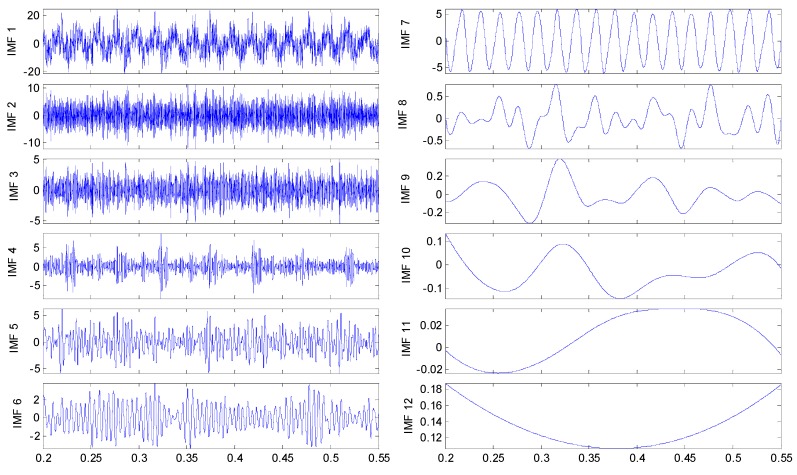
IMFs for the axle bearing vibration signal with cage fault.

**Figure 8:**
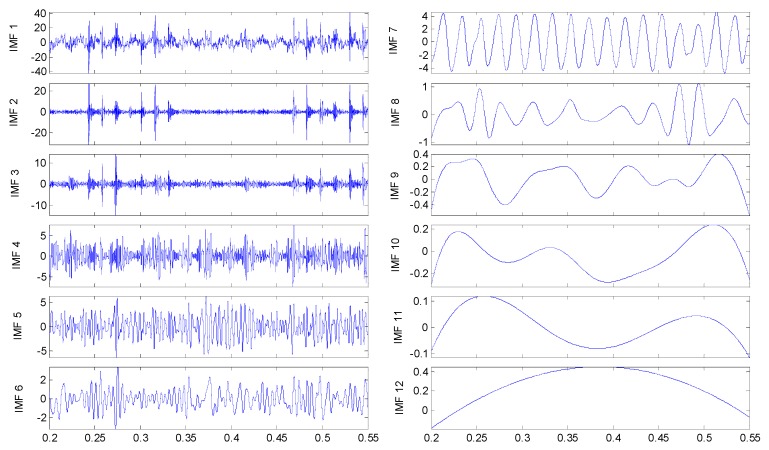
IMFs for the axle bearing vibration signal with pin roller fault.

To discriminate the four conditions, the EEMD is applied to all of these original signals: there are 12 IMFs decomposed in total for the four vibration signals, and the twelfth IMF is the residue on behalf of the trend. All the IMFs extracted are reported in Figure 5, Figure 6. Figure 7 and Figure 8, respectively.

By examining the functions resulting from the decomposition, it appears that, independently from the axle bearing condition, the first IMFs describe high frequency phenomena while the last one is related to the low frequency components of the signals that could have not physical meaning and could be due to the stop criteria set in the sifting process. Considering that the fault signature is related to the rotation frequency, very low frequency components should not be of interest. In any case the selection of the IMFs on the basis of the IMFs’ confidence index algorithms allows these spurious IMFs to be always and automatically discarded from the set used for any future analysis. The one-to-one comparison between IMFs of the same order highlights differences in both shape and amplitude depending on the health condition. However, the fault detection is quite difficult in spite of the visible differences of the IMF features between the pin roller fault and the others: hidden variations that could be related to the fault can be traced in the decompositions, even if in different IMFs. Obviously, in accord with the previous remarks, not all the IMFs are considered for fault diagnostics.

When the faults occur in the bearing, the energy of the bearing vibration signals would change strongly in local frequency bands, while in other frequency bands the energy maybe change weakly. Analyzing the whole process of the Hilbert transform for the IMFs, it is obvious that both frequency and amplitude of each IMF are the function of time, meaning the Hilbert instantaneous frequency spectrum, which offers a measure of amplitude distribution with change of every time and frequency, while the marginal spectrum gives a measure of the total amplitude distribution from each frequency. On another hand, the local damage in the bearing will always generate the corresponding fault characteristic frequency, and the corresponding energy of this frequency band will also change, which can be presented distinctly by the amplitude of the marginal spectrum. Therefore, this is a feasible way to use the marginal spectrum to capture the fault characteristic frequency.

**Figure 9:**
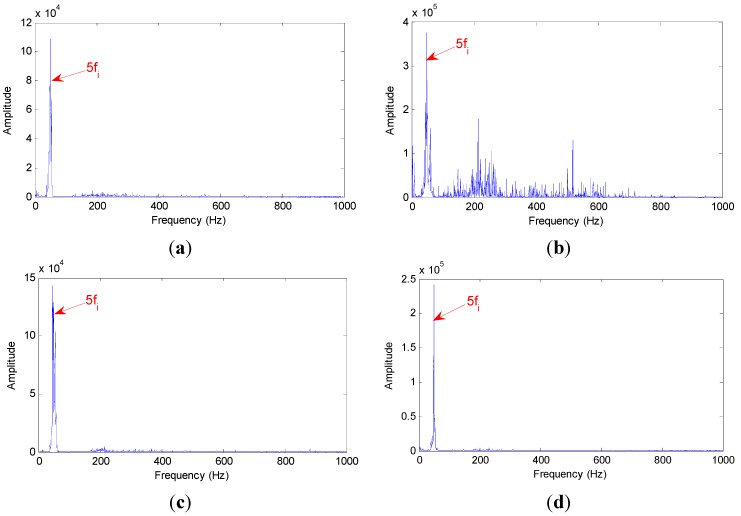
Hilbert marginal spectrum of all IMFs for axle bearing vibration: (**a**) undamaged bearing; (**b**) outer ring fault bearing; (**c**) cage fault bearing; (**d**) pin roller fault bearing.

The Hilbert marginal spectrums of all IMFs of axle bearing vibration signals under four working conditions are calculated for the comparison with the minority IMFs selected by the proposed method, showed as Figure 9a–d, respectively. The Hilbert marginal spectrum represents the fluctuation of the energy distribution of axle bearing vibration with frequency, and the larger the amplitude, the more the energy distribution of the frequency band is. There is a main frequency peak approximating 51 Hz, which is consistent in each marginal spectrum in Figure 9, and corresponds to the five multiplier rotation frequency of the axle bearing at the wheel speed of 100 km/h.

Figure 9 shows that the Hilbert marginal spectrum can extract the fundamental and multiplier frequency of rotation effectively, but this way calculating with all IMFs leads to the fault features submerging in fundamental frequency of vibration signal. The core target of IMF’s confidence index algorithm is to eliminate the effect of the overlap frequency that submerges fault features and highlight the failure characteristics.

According to Section 3, the confidence index for each IMF is evaluated: the values assumed by the parameter for the four health condition axle bearings are reported in Table 2, Table 3, Table 4 and Table 5, respectively. These positive values of confidence index based on the self-correlation quality screening are obtained: IMFs 1, 5 and 9 are selected for the undamaged axle bearing; IMFs 4, 5 and 6 are selected for the axle bearing with outer ring fault; only IMF 8 is selected for the axle bearing with cage fault; and IMFs 6 and 8 are selected for the axle bearing with pin roller fault. These are the IMFs chosen by the IMFs’ confidence index algorithm and subjected to further analysis, to verify whether these IMFs provide enough confidence to diagnose the faults.

**Figure 10:**
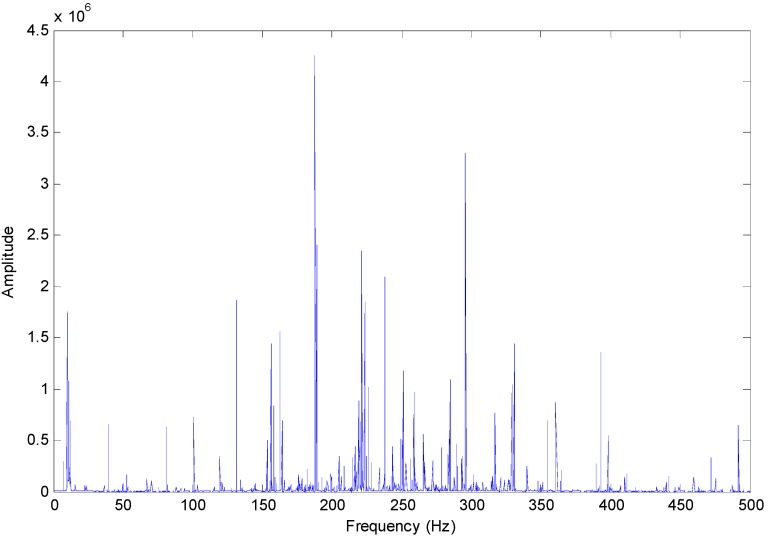
Hilbert marginal spectrum of selected IMFs for the undamaged axle bearing vibration signal.

**Figure 11:**
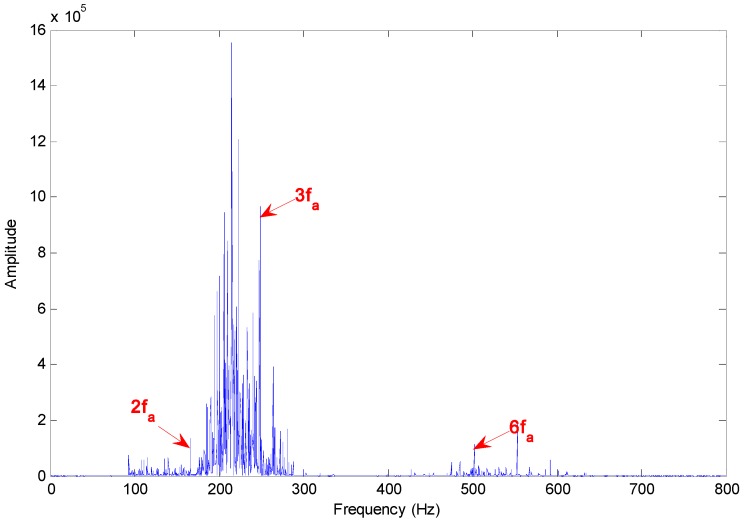
Hilbert marginal spectrum of selected IMFs for axle bearing vibrations with outer ring faults.

**Figure 12:**
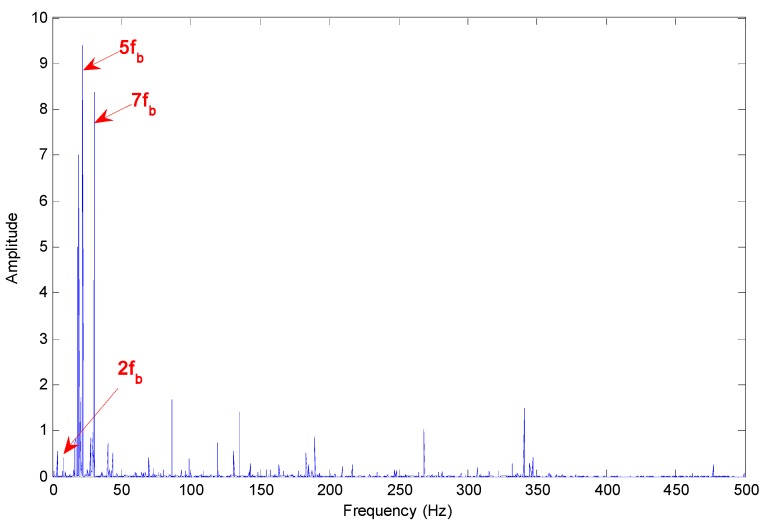
Hilbert marginal spectrum of selected IMFs for axle bearing vibrations with cage faults.

**Figure 13:**
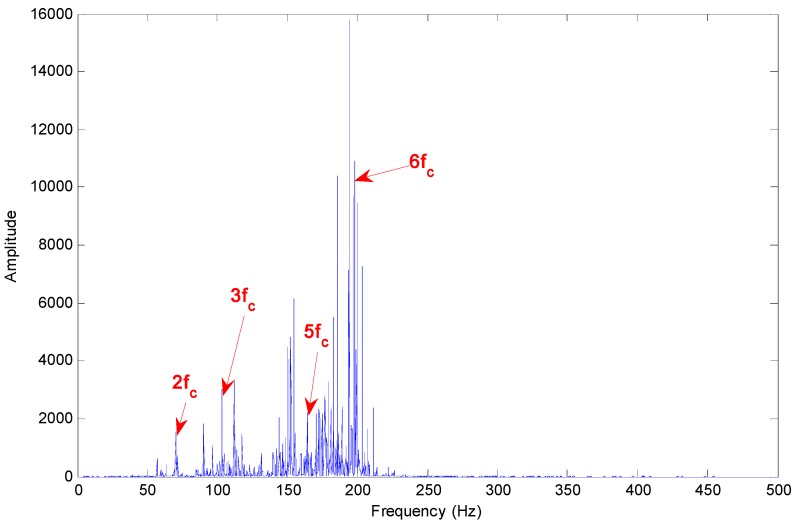
Hilbert marginal spectrum of selected IMFs for axle bearing vibrations with pin roller faults.

The Hilbert marginal spectra of axle bearing vibration signals for four working conditions with different faults are shown in Figure 10, Figure 11, Figure 12 and Figure 13, respectively. Figure 10 shows the Hilbert marginal spectrum of selected IMFs for undamaged axle bearing vibration signal. There are multiple frequency peaks, and the main frequency band cannot be found. This can be explained by the fact that the energy distribution is uniform in the frequency direction when there is nothing damaged on the axle bearing.

**Table 2:** IMFs’ confidence index calculation for the undamaged axle bearing vibration.

IMF	Sc. Quality	S. Absolute	K. Characteristics	I. Allowance	C. Index	E. Result
1	0.1233	0.1219	0.1009	0.0168	0.0378	√
2	0.0361	0.0107	0.1607	0.0787	−0.0712	×
3	0.0382	0.0119	0.0248	0.0860	0.0731	×
4	0.0941	0.0208	0.0638	0.0479	0.0048	×
5	0.1571	0.1554	0.0995	0.0426	0.0985	√
6	0.1290	0.0531	0.1203	0.0489	−0.0183	×
7	0.3991	0.0499	0.4197	0	−0.3698	×
8	0.1830	0.0375	0.0750	0	−0.0375	×
9	0.3238	0.2692	0.2642	0	0.0050	√
10	0.2757	0.1138	0.1764	0	−0.0625	×
11	0.3774	0.2020	0.3620	0	−0.1600	×

**Table 3:** IMFs’ confidence index calculation for the axle bearing vibration with outer ring fault.

IMF	Sc. Quality	S. Absolute	K. Characteristics	I. Allowance	C. Index	E. Result
1	0.1029	0.0162	0.0979	0.0183	−0.0634	×
2	0.0334	0.0189	0.1286	0.1570	0.0473	×
3	0.0775	0.0297	0.0195	0.0463	0.0565	×
4	0.1310	0.0569	0.1092	0.0555	0.0032	√
5	0.2247	0.0546	0.1315	0.0794	0.0024	√
6	0.1739	0.1301	0.1365	0.1624	0.1559	√
7	0.3459	0.2384	0.2909	0	−0.0524	×
8	0.2806	0.0119	0.2031	0.1812	−0.0100	×
9	0.2142	0.1108	0.1433	0	−0.0325	×
10	0.3421	0.2046	0.2077	0	−0.0031	×
11	0.4201	0.1138	0.3385	0	−0.2247	×

**Table 4:** IMFs’ confidence index calculation for the axle bearing vibration with cage fault.

IMF	Sc. Quality	S. Absolute	K. Characteristics	I. Allowance	C. Index	E. Result
1	0.1386	0.0893	0.1152	0.0126	−0.0132	×
2	0.0338	0.0117	0.1891	0.0428	−0.1346	×
3	0.0397	0.0235	0.1146	0.0760	−0.0151	×
4	0.0902	0.0217	0.0421	0.0538	0.0334	×
5	0.1528	0.0323	0.1248	0.0614	−0.0311	×
6	0.2273	0.0386	0.1706	0.1042	−0.0279	×
7	0.3987	0.0316	0.4159	0	−0.3843	×
8	0.2037	0.2907	0.1249	0.3760	0.5417	√
9	0.2794	0.0631	0.0783	0	−0.0153	×
10	0.2745	0.2204	0.2357	0	−0.0153	×
11	0.4293	0.2687	0.4586	0	−0.1899	×

**Table 5:** IMFs’ confidence index calculation for the axle bearing vibration with pin roller fault.

IMF	Sc. Quality	S. Absolute	K. Characteristics	I. Allowance	C. Index	E. Result
1	0.1108	0.0115	0.0229	0.0102	−0.0013	×
2	0.0366	0.6663	0.0011	0.0169	0.6822	×
3	0.0407	0.1776	0.0034	0.0365	0.2107	×
4	0.0788	0.0131	0.0812	0.0495	−0.0186	×
5	0.1503	0.0234	0.1473	0.0686	−0.0553	×
6	0.1192	0.1695	0.1241	0.0859	0.1313	√
7	0.3899	0.0323	0.3718	0	−0.3395	×
8	0.1720	0.1565	0.1170	0.3013	0.3408	√
9	0.2833	0.1551	0.1840	0	−0.0289	×
10	0.2775	0.0842	0.2428	0	−0.1586	×
11	0.3230	0.2157	0.2698	0	−0.0541	×

Figure 11 shows the Hilbert marginal spectrum of selected IMFs for axle bearing vibration signals with outer ring faults, where the main frequency band is mainly concentrated at approximately 200 Hz; nonetheless the frequency multiples of the characteristic fault frequency are quite evident: the fault characteristic frequency of the outer ring fault for the axle bearing is 3 × *f_a_* in this experimental test, and there are obvious frequency peaks corresponding to the frequencies of 2 × *f_a_*, 3 × *f_a_* and 6 × *f_a_*.

Figure 12 presents the Hilbert marginal spectrum of selected IMFs for axle bearing vibration signals with cage faults, where the main peaks correspond to the frequencies of 2 × *f_b_*, 5 × *f_b_* and 7 × *f_b_*. The cage fault diagnosis result is a quite gratifying.

Figure 13 presents the Hilbert marginal spectrum of selected IMFs for axle bearing vibration signals with pin roller faults. There is a mass of sidebands around the several main frequency peaks, but the frequency multiples of the fault characteristic frequency are well-marked and substantive: 2 × *f_c_*, 3 × *f_c_*, 5 × *f_c_* and 6 × *f_c_*.

Due to the bearing rolling features and the fault characteristics, it is impossible to cause a sort of “regularization” of the peaks, *i.e.*, the relative increase of the components due to the fault with respect to the components due to the rotation and with a period related to fault presence, which is described in [3]. According to Figure 11, Figure 12 and Figure 13, the fault characteristic frequency and different fault diagnostics results are highlighted by the Hilbert marginal spectrum with concentration and intuitive effect. With a comparison of the fault diagnosis ability of the Hilbert marginal spectrum using all the IMFs and selected IMFs by the presented method, the latter has quite a high presentation in spectral resolution and failure signature.

**Figure 14:**
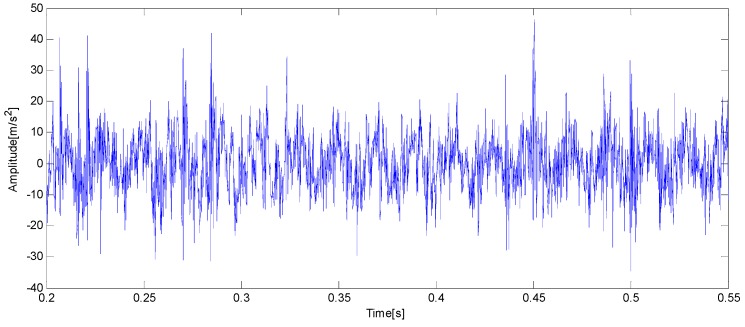
Vibration signal of an axle bearing with a composite of three kinds of faults.

From what has been discussed and analyzed above, using the IMF’s confidence index and Hilbert marginal spectrum to diagnose axle bearing faults is an advanced algorithm and signal processing technique. Since no hypothesis about the stationary and the periodicity of the signal must be respected for EEMD and marginal spectrum applications, the presented approach is tested on data collected for an axle bearing with a specific and single fault. The next question is whether the algorithms and signal processing by the IMF’s confidence index and Hilbert marginal spectrum can maintain their usefulness in a combined faults test. The vibration signal of an axle bearing with a composite of three kinds of faults: outer ring fault, cage fault and pin roller fault, as described as Figure 3a–c, is shown in Figure 14.

**Figure 15:**
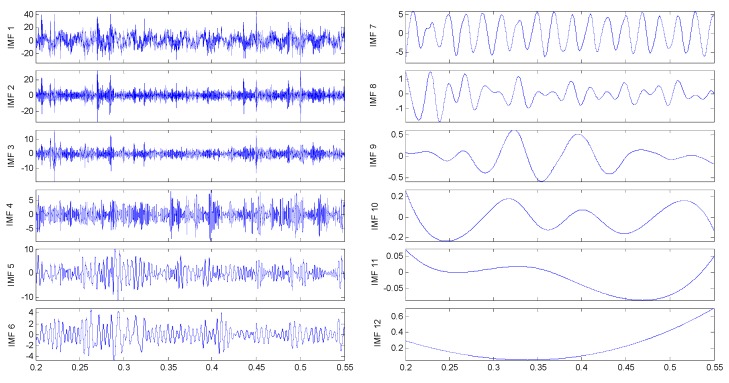
IMFs for the axle bearing vibration signal with a composite of three kinds of faults.

EEMD is applied and the corresponding IMFs extracted for this axle bearing vibration signal are reported in Figure 15. The fault signature is already apparent at this stage: the fifth and sixth IMFs are affected by a modulated amplitude variation.

**Table 6:** IMFs’ confidence index calculation for axle bearing vibration with a composite of three kinds of faults.

IMF	Sc. Quality	S. Absolute	K. Characteristics	I. Allowance	C. Index	E. Result
1	0.0906	0.1622	0.0621	0.0087	0.1088	×
2	0.0283	0.1265	0.0212	0.0165	0.1219	×
3	0.0339	0.0245	0.0151	0.0263	0.0357	×
4	0.0711	0.0276	0.0766	0.0538	0.0048	×
5	0.1364	0.0777	0.0782	0.0327	0.0323	√
6	0.1362	0.1419	0.1002	0.0765	0.1182	√
7	0.3832	0.0513	0.3657	0	−0.3144	×
8	0.2034	0.0616	0.0754	0	−0.0138	×
9	0.2875	0.1360	0.1090	0	0.0269	√
10	0.2999	0.0208	0.2815	0	−0.2607	×
11	0.3371	0.2812	0.2851	0	−0.0039	×

**Figure 16:**
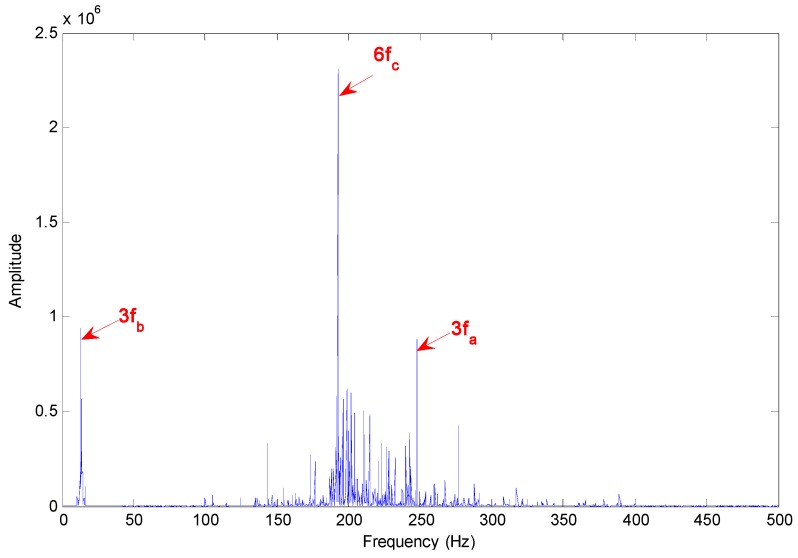
Hilbert marginal spectrum of selected IMFs for axle bearing vibration signals with a composite of three kinds of faults.

Based on the IMF’s confidence index arithmetic, the IMFs 5, 6 and 9 selected and evaluated can diagnose the faults with a high degree of confidence showed in Table 6. The Hilbert marginal spectrum has also been estimated in this case. Its energy distribution with frequency is shown in Figure 16. The presence of the faults is evident by that where the main peaks lie corresponds to the frequencies of 3 × *f_a_*, 3 × *f_b_* and 6 × *f_c_*, which represent the outer ring fault, cage fault and pin roller fault, respectively.

The diagnosis result for composite fault detection by means of the Hilbert marginal spectrum is more gratifying, which seems more evident for the last case considered, with composite faults, than for the previous one. This remark is very important, above all from an operational point of view: the robustness and the diagnostics effectiveness of this arithmetic and technique make it very useful for on-line diagnostics of railway axle bearings. Based on the EEMD method as a data-based decomposition and Hilbert marginal spectrum as the energy distribution evaluation method with frequency, it is possible that the positive results obtained from practical axle bearing fault detection applications can be still improved for more complex mechanical systems.

## 6. Conclusions

The aim of the paper was the improvement of EEMD and the Hilbert marginal spectrum by means of the introduction of an IMF’s confidence index that allows the adaptive self-selection of the IMFs. Indeed, not all the IMFs obtained by the decomposition should be considered in the Hilbert marginal spectrum. The IMF’s confidence index arithmetic is fully adaptive, overcoming the major limitation of selection by users with experience, and allows the development of on-line tools. Of course, the automatic identification of characteristic fault frequencies in the final diagnostic step based on the Hilbert marginal spectrum still needs improvement. The proposed adaptive selection allows successful diagnosis of railway axle bearings with different faults: outer ring faults, cage faults and pin roller faults. Whether for a single fault or multiple composite faults, a satisfactory diagnosis result is always obtained. In this way, the powerful characteristics of the EEMD and Hilbert marginal spectrum are fully exploited.
